# Preoperative Bowel Preparation in Minimally Invasive and Vaginal Gynecologic Surgery

**DOI:** 10.1155/2020/8546037

**Published:** 2020-02-14

**Authors:** Michail Diakosavvas, Nikolaos Thomakos, Alexandros Psarris, Zacharias Fasoulakis, Marianna Theodora, Dimitrios Haidopoulos, Alexandros Rodolakis

**Affiliations:** 1st Department of Obstetrics and Gynaecology, “Alexandra” Hospital, National and Kapodistrian University of Athens, Athens, Greece

## Abstract

Bowel preparation traditionally refers to the removal of bowel contents via mechanical cleansing measures. Although it has been a common practice for more than 70 years, its use is based mostly on expert opinion rather than solid evidence. Mechanical bowel preparation in minimally invasive and vaginal gynecologic surgery is strongly debated, since many studies have not confirmed its effectiveness, neither in reducing postoperative infectious morbidity nor in improving surgeons' performance. A comprehensive search of Medline/PubMed and the Cochrane Library Database was conducted, for related articles up to June 2019, including terms such as “mechanical bowel preparation,” “vaginal surgery,” “minimally invasive,” and “gynecology.” We aimed to determine the best practice regarding bowel preparation before these surgical approaches. In previous studies, bowel preparation was evaluated only via mechanical measures. The identified randomized trials in laparoscopic approach and in vaginal surgery were 8 and 4, respectively. Most of them compare different types of preparation, with patients being separated into groups of oral laxatives, rectal measures (enema), low residue diet, and fasting. The outcomes of interest are the quality of the surgical field, postoperative infectious complications, length of hospital stay, and patients' comfort during the whole procedure. The results are almost identical regardless of the procedure's type. Routine administration of bowel preparation seems to offer no advantage to any of the objectives mentioned above. Taking into consideration the fact that in most gynecologic cases there is minimal probability of bowel intraluminal entry and, thus, low surgical site infection rates, most scientific societies have issued guidelines against the use of any bowel preparation regimen before laparoscopic or vaginal surgery. Nonetheless, surgeons still do not use a specific pattern and continue ordering them. However, according to recent evidence, preoperative bowel preparation of any type should be omitted prior to minimally invasive and vaginal gynecologic surgeries.

## 1. Introduction

Bowel preparation (BP) before surgery traditionally refers to the removal of bowel contents via mechanical cleansing with oral or rectal mechanical measures. Despite the fact that administration of preoperative bowel preparation has been a common practice for more than 70 years, its use is based mostly on expert opinion rather than solid evidence [1–3]. The proposed benefits from the use of bowel preparation include reduced rates of surgical site infections (SSI), easier manipulation of the bowel during surgery, and reduced rates of anastomotic leakage (AL) in case of bowel anastomosis [4, 5]. However, despite the lack of supporting literature, mechanical bowel preparation (MBP) still represents an ingrained practice before gynecologic surgery as in other surgical specialties [5, 6].

Oral antibiotic bowel preparation (OABP), which constitutes another aspect of BP, has emerged during the last decades in order to address the need of further reducing patients' postoperative morbidities and mortality. This comes as a result of the decrease of intraluminal bacterial load and, subsequently, of SSI rates, in case of bowel injury [2, 3].

Preoperative BP regimen in minimally invasive gynecologic surgery is debated. A great number of studies investigating the effect of preoperative BP in laparoscopic operations have not confirmed its effectiveness, neither in reducing postoperative infectious morbidity nor in improving surgeons' performance. Nonetheless, supporters of MBP still suggest that an empty bowel results in a better surgical view and a less contaminated surgical field [4, 6–12].

The use of MBP in vaginal surgery is even more controversial. Only four randomized controlled studies exist in the literature, one of which refers to a combined laparoscopic and vaginal approach. All evidence is against the use of MBP in vaginal surgery [13–16]. However, as with laparoscopic surgery, MBP is still used by many surgeons who appear reluctant to omit it from day to day practice, despite the fact that scientific societies have addressed the need of specific guidelines, by issuing recommendations against the use of MBP solely.

## 2. Methods and Objectives

We conducted a comprehensive search of the PubMed/Medline and the Cochrane Database using the following terms: bowel preparation, intestinal preparation, and mechanical bowel preparation with minimally invasive gynecologic surgery and vaginal surgery, and related articles from the latest two decades up to June 2019 were scanned for relevance. We applied no restriction to region or publication type. Manuscripts published in any language other than English were excluded from our study. Abstracts were scanned for relevance from DM and PA, before appraising the full-text articles. The reference lists of all eligible published articles were crosschecked by FZ, TM, and HD. Manuscripts were selected by consensus of DM, PA, and FZ for a complete review and any uncertainties were resolved by consensus discussion with the senior author (TN). This literature was summarized by one author (DM) and sent to an internal expert author for review (RA).

The purpose of this review was to evaluate the effect of bowel preparation before laparoscopic or vaginal surgery on bowel manipulation, surgical field view, operative time, SSI rate, duration of hospitalization, morbidity, and also patients' and surgeons' satisfaction. The detection of all available existing guidelines from different scientific institutions and the surgeons' preferred and commonly ordered practices constituted a part of our objectives. Hence, we aimed to determine the best practice regarding bowel preparation before minimally invasive and vaginal gynecologic surgery.

## 3. Results

### 3.1. Bowel Preparation before Minimally Invasive Gynecologic Surgery

In the field of BP in minimally invasive gynecologic surgery, most studies have specifically evaluated the use of mechanical BP measures.

One of the main arguments, of those in favor of MBP, is the improvement of surgical field's visibility and intraoperative bowel handling [8, 17]. Laparoscopic surgeons' choice of MBP as a preoperative standard is based on the idea that the empty bowel will occupy less space allowing for better carbon dioxide insufflation of the abdomen and, hence, a better view [4]. MBP use is also supported by the belief that it reduces the prevalence of fecal contamination in case of inadvertent bowel injury or scheduled bowel resection, via decreasing the bacterial load [4, 8, 9]. As a result, it is believed that MBP protects against complications, such as surgical site infections, anastomotic leakage, and fecal peritonitis by minimizing the fecal load of the bowel [6, 11, 12]. On the other hand, it is argued that laparoscopic surgery can be facilitated by the presence of solid matter inside the colon in order for gravity to help get a better view of the peritoneal cavity [7]. Furthermore, some studies suggest that MBP could actually increase the risk of anastomotic leakage, due to bowel irritation caused by the laxatives [10].

Most randomized controlled trials (RCTs) compare oral MBP to no preparation regimen, besides fasting or a type of low residue diet [17–21]. Fewer studies compare oral MBP to enema MBP [11, 13]. Only a small part of them compare enema use to no MBP [8, 12] (Table 1).

It must be taken into account that some of the studies mentioned above exclude patients with suspected or anticipated malignancy, or with severe endometriosis, mainly in the cul de sac, because of possible enteric resection [8, 11–13, 17–20]. Similarly, obese patients that might require an advanced laparoscopic procedure and even patients with prior surgery to the pelvis or the abdomen did not meet the inclusion criteria [18, 19].

One of the very first randomized controlled trials studying different types of preoperative bowel preparation in gynecology compares MBP with 90 mL oral solution of sodium phosphate vs no MBP in laparoscopy [18]. The results were in agreement with previously conducted trials with similar objectives in colorectal surgery [22]. While there was no significant difference from the surgeons' point of view (same operative time and difficulty, comparable scores concerning surgical field view), patients' experience was not the same between the 2 groups. In the MBP group, significantly higher discomfort was observed preoperatively, mainly because of insomnia, weakness, abdominal distension, hunger, thirst, nausea, and vomiting, in contrast to the no MBP group [18].

On the other hand, in an RCT by Won et al., which investigated the same parameters, intraoperative surgical exposure and bowel handling have been shown to be statistically better in patients receiving MBP (oral sodium picosulfate) compared to those who either only fasted prior to laparoscopy or received a minimal residue diet for 2 days before the operation. Despite these results, the fasting-only approach was recommended after taking into consideration the distressfulness and the adverse effects of MBP on women [17]. Unlike Won et al., Bakay and Aytekin in 2017 investigated the field of vision and surgical comfort during total laparoscopic hysterectomy procedures in 102 patients, using a visual indexing tool based on anatomic landmarks and found no differences in surgical view or intraoperative time between the group receiving oral MBP and the only fasting one (mean operation time: oral sodium phosphate (NaP) group (47.42 min) vs no MBP group (48.54 min), *p*=0.847) [21].

In 2009, Lijoi et al., instead of evaluating MBP vs no MBP, compared a 7-day low fiber intake vs MBP consisting of four doses of a granular powder dissolved in 1,000 mL of water per dose, in gynecologic laparoscopic procedures. They reached similar conclusions as previously conducted studies, showing no difference in surgical field exposure, higher tolerance, and less discomfort preoperatively in the low fiber intake group compared to the MBP group. Surgical time was comparable between the two groups, as was the length of hospital stay (LOS) [19].

Several other studies have concluded that MBP can be well tolerated by patients preoperatively without any major discomfort, but as previously shown, according to the surgeons' point of view, no statistically significant difference was found regarding the surgical field between women who underwent MBP and those who did not. Interestingly, surgeons were able to correctly predict whether the patient was administered MBP or not, only in 55–59% of the cases [8, 20].

In contrast to previous studies, Yang et al. (2011) compared efficiency of oral MBP vs enema MBP with NaP in advanced gynecologic laparoscopic procedures without very strict exclusion criteria. For instance, cases with obesity, history of previous surgery, and more complicated laparoscopic surgeries (such as excision of endometriosis with or without presacral neurectomy) were all included in the study. In accordance with previous literature, surgeons' assessment of surgical field showed no difference between the two groups (graded as excellent or good in 85% of patients in oral MBP and in 91% in enema group), resulting in similar surgical time and difficulty. Patients in the oral MBP group reported a significantly more unpleasant experience than those in the enema MBP group due to symptoms of abdominal swelling, nausea, and dizziness. Many of them stated that in case they had to undergo a surgical procedure again, they would choose a different preoperative bowel preparation type [11].

Preparation with enema use does not seem to be more effective when compared to fasting. In gynecologic laparoscopic surgery, using MBP in a form of either oral regimen or enema does not improve intraoperative visualization of surgical field, nor bowel handling. This also applies to cases where a large uterus is to be removed or to patients with higher BMI. Oppositely, overall discomfort of patients is anticipated to be much less, when fasting is the type of preoperative BP, compared to patients who undergo some form of MBP—oral or enema [12].

### 3.2. Bowel Preparation before Vaginal Surgery

To our knowledge, there are only four randomized control studies, investigating the use of BP prior to vaginal surgery (Table 2).

Ballard et al. studied surgeons' intraoperative assessment as well as patients' satisfaction. Women were divided into 2 groups, one receiving MBP with two saline enemas and the other not receiving anything per os after midnight before surgery. Women underwent vaginal prolapse surgery with apical suspension and posterior colporrhaphy. Discomfort for women in the enema group was significantly higher, with hunger, weakness, abdominal swelling, and anal irritation being the most common causes, resulting in statistical difference in patients' complete satisfaction between the two groups (66% in saline enema group vs 94% in no MBP group, *p* < 0.001). On the other hand, no difference was found regarding surgeons' assessment of bowel content and surgical site visualization [14].

Adelowo et al. compared the use of MBP (using oral magnesium citrate combined with sodium phosphate enema) to sodium phosphate enema alone, during minimally invasive pelvic reconstructive surgery. The MBP group reported greater overall discomfort and more side effects than the enema-only group. The quality of the surgical field was the same when appreciated in the conclusion of the operation, despite an initial advantage of the MBP group during port placement. Return of bowel function was the same in both groups (2–4 days, median 3 days) [13].

More recently (2019), a randomized single blind comparison of bowel preparation regimens for pelvic organ prolapse was conducted. Among 60 patients who received polyethylene glycol orally and 60 patients with no bowel preparation preoperatively, no difference was found regarding the cleansing of surgical field. Conversely, adverse effects were significantly higher in the group of patients with intestinal preparation while abdominal distention was reported by 22% and nausea by 8% of patients in the MBP group [13].

Moreover, the use of MBP in vaginal prolapse surgical treatment is being discouraged by another RCT by Tayyab et al. When patients' response was assessed postoperatively via evaluation of their symptoms (nausea, vomiting, and anal irritation), no difference was reported among patients treated preoperatively with saline enemas and those with regular diet. Therefore, they concluded that there is no need for preoperative hospitalization for the purpose of presurgical MBP [16].

## 4. Discussion

The necessity of MBP in gynecologic surgery has been under investigation for the last decade, regarding its benefits, its possible side effects, and its effectiveness when compared to other types of preoperative bowel preparation. Many meta-analysis and reviews have been published regarding MBP's efficacy and its possible side effects. Most authors set as a primary outcome of interest the quality of the surgical field, postoperative complications, and patients' comfort during the whole procedure, but they also set secondary objectives like length of hospital stay (LOS) and economic costs [23]. The results are almost identical. Regardless of the type of procedure (laparoscopy, robotic, or vaginal surgery), routine administration of MPB seems to offer no advantage to any of the objectives mentioned above [24–26]. Surgical field visualization is irrelevant to the type of preoperative bowel preparation [25], bowel handling is the same whether MBP is used or not, surgical site infection rates are not affected by MBP use [26], LOS is not increased when MBP is omitted [9, 19], while patients' discomfort and adverse physiologic effects are significantly higher when oral laxatives are used [24, 25].

The most common MBP regimens include the use of laxatives which are administered either orally or rectally [24, 25]. Sodium phosphate (NaP) can be used either as an enema or as an oral preparation, while polyethylene glycol is used orally. Other laxatives such as lactulose, sorbitol, glycerin, ducosate, bisacodyl, or castor oil are scarcely prescribed [1].

The negative effects of bowel preparation include patients' discomfort, such as postoperative pain, nausea, vomiting, abdominal distension, insomnia, weakness, and various physiologic changes [24, 25]. BP with bisacodyl and sodium phosphate resulted in severe dehydration which led to a significant decrease in exercise capacity and weight. Moreover, an increase in phosphate, urea serum concentrations, and plasma osmolality combined with a significant drop of serum calcium and potassium was also observed [27]. Similar metabolic disturbances can result from the use of phosphate enema alone, as described by Mendoza et al. [28].

Evidently, the use of mechanical bowel preparation is of little to no use in minimally invasive and vaginal gynecologic surgery. Hence, most surgical scientific organizations have issued guidelines against the use of MBP. WHO SSI prevention guidelines, NICE guidelines of 2019, the guidelines of the American Society of Colon and Rectal Surgeons (ASCRS), and those of the Canadian Society of Colon and Rectal Surgeons (CSRS), the RCOG and the ACOG, advise against the sole use of mechanical bowel preparation [29–34]. To our knowledge, the use of MBP is not recommended by any scientific body, prior to minimally invasive or vaginal gynecologic surgery.

However, MBP may be acceptable only in combination with oral antibiotics bowel preparation. Recent evidence from a great number of studies has suggested that the combined use of MBP with OABP may have a beneficial effect on reducing postoperative complication rates (SSI, AL, and LOS) and eventually patients' morbidity. The effectiveness of combined BP is more obvious, when applied preoperatively, in surgical operations with high probability of intraluminal entry, resulting in less sterile surgical field, such as in colorectal surgery and in gynecologic procedures of high complexity [2, 3, 29]. Therefore, some scientific societies that take these recent data into account have issued complimentary recommendations suggesting that MBP use in conjunction with OABP is the safer approach, at least in colorectal surgery [29, 31].

Unlike colorectal operations, in gynecologic and gynecologic oncology surgeries, bowel entry represents an uncommon phenomenon; however, bowel involvement either planned (bowel/colon resection) or iatrogenic (injury) can complicate either cases of advanced ovarian cancer, where cytoreductive surgery is required, or cases of severe endometriosis [4]. In most of the rest gynecologic cases, patients' morbidity remains low. Kafy et al. in an audit of 1792 hysterectomies for benign, nonobstetric reasons showed that the overall morbidity rate for laparoscopic approach was 9.4%, with only 0.4% being attributed to bowel injury, while in vaginal approach these percentages were 8.7% and 0%, respectively [35]. Readmission and overall infection rates were similarly low in both types of surgery (0.9% and 0.89% in minimally invasive cases, 1.3% and 0.9% in vaginal surgeries). In other reviews, the incidence of laparoscopy-induced gastrointestinal injury has even been reported to be as low as 0.13% and that of bowel perforation 0.22% [36].

In 2019, Kalogera et al. reported that BP does not offer any protection against SSI and, overall, against postoperative infectious morbidity in minimally invasive gynecologic surgery, regardless of the type of preparation used and, thus, it could be safely abandoned [37]. As it is easily understood, in gynecologic cases with minor probability of bowel resection or injury and low SSI rates, preoperative BP, mechanical, or oral antimicrobial agents do not offer any major advantage. On that account, Enhanced Recovery After Surgery (ERAS) Society in 2019 updated their recommendations regarding preoperative bowel preparation. Routine preoperative BP is strongly discouraged before minimally invasive gynecologic surgery; they suggest that the combination of MBP and OABP, or even oral antibiotics alone, should only be considered when colon resection is planned [38].

In a survey published in 2005, regarding bowel preparation before abdominal surgical treatment of malignancy which included a resection of the colon, oral cleansing was by far the rule among surgeons asked [39]. Questionnaires addressed specifically to surgeons of gynecologic laparoscopy, or of vaginal procedures, could not be traced, due to lack of relevant literature. Nonetheless, MBP sole use, as previously mentioned, is not recommended by any scientific society in any surgical approach. However, colorectal surgeons across the globe, still, do not follow a specific pattern or evidence-based current recommendations. In 2018, in another survey that was conducted among members of the Chinese Society of Colorectal Cancer (CSCC), at least 50% of surgeons admitted administering MBP use exclusively [40]. Based on our experience, it would be completely justified to extract data from other surgical specialties and recognize the fact that gynecologists also continue using MBP regimens in laparoscopic or vaginal surgical procedures, despite the updated data and extensive literature suggesting otherwise.

## 5. Conclusion

MBP is common not only in laparotomy but also in minimally invasive and vaginal surgery. Recently, the use of oral antibiotics for bowel preparation before surgery has emerged as an adjunct to MBP, aiming to reduce the high rates of postoperative septic complications and patients' morbidity with or without MBP, mostly in colorectal surgery. Nonetheless, they have not been widely accepted and have not been fully implemented in day to day practice. Despite the theoretical advantages of MBP, most available studies, either in gynecologic laparoscopy or in vaginal surgery, conclude that MBP does not reduce SSI rates, does not improve bowel manipulation, field of view, and operating time, and does not affect patients' morbidity. On the other hand, MBP has a negative psychological and physiological impact on patients. Hence, MBP represents a point of debate for the scientific community that even led physicians and scientific committees to provide criteria and issue guidelines for excluding bowel preparation prior to specific types of surgery by highlighting the operations with minimal possibility of enteric participation. In particular, in the case of laparoscopic and vaginal gynecologic surgery, the risk of bowel intraluminal entry because of an injury or a planned enteric resection is minor. Consequently, the need of bowel anastomosis, SSI rate, and overall postoperative morbidity and mortality rate is also minimal. Therefore, according to recent evidence, preoperative bowel preparation of any type, mechanical or oral antibiotics, should be omitted prior to these surgical approaches.

## Figures and Tables

**Table 1:** Studies assessing the use of bowel preparation in minimally invasive gynecologic surgery.

Study (reference)	Regimen of BP compared (group size/no. of patients)			Outcomes of interest and results
Muzii et al. [18]	MBP-oral NaP (81)	No MBP (81)		Greater patients' discomfort in the MBP group No difference in surgeons' evaluation of the surgical field, operative difficulty, operative time, and postoperative complications

Lijoi et al. [19]	MBP-oral granular powder dissolved in 1000 mL (41)	1-week low fiber diet <10 g (42)		No difference in evaluation of surgical field and operative time Abdominal distension and overall discomfort were more frequent in MBP group No difference in postoperative pain, nausea, abdominal swelling, ileus rate, and LOS

Yang et al. [11]	MBP-oral NaP (72)	MBP-NaP enema (73)		No difference in evaluation of the surgical field, bowel handling, degree of bowel preparation, or surgical difficulty Abdominal bloating and swelling, weakness, thirst, dizziness, nausea, fecal incontinence, and overall discomfort were greater in the oral solution group

Won et al. [17]	Minimal residue diet + MPB-oral Na picosulphate (87)	Minimal residue diet (84)	Fasting only (86)	Better surgical view with minimal residue diet + MBP No difference in complications Greater patients' symptoms in MBP group (headache, thirst, weakness, tiredness, and overall discomfort) by VAS

Siedhoff et al. [8]	MBP-single NaP enema (73)	No MBP (73)		No difference in anxiety by VAS No difference in evaluation of surgical field Same operative time and blood loss No difference in postoperative constipation or patients' rating of symptoms (cramps, hunger, bloating, embarrassment, weakness, dizziness, thirst, nausea, incontinence, and constipation) Increased insomnia in no MBP group

Ryan et al. [20]	MBP-oral magnesium citrate (39)	No MBP (39)		No difference in intraoperative visualization, bowel handling, or overall ease of the operation Same compliance, preoperative and postoperative patients' discomfort

Bakay and Aytekin [21]	MBP-oral NaP (NR)	No MBP (NR)		No difference in operative time

Mulayim and Karadag [12]	MBP-oral NaP (96)	MBP-enema NaP (92)	Fasting only (90)	No difference in visualization of the surgical field, ease of bowel handling, and overall ease of surgery based on VAS score No benefit of MBP when removing large uteri or when operating on patients with a high BMI Preoperative overall discomfort score was better in the fasting-only group

MBP = mechanical bowel preparation; NaP = sodium phosphate; NR = not reported; LOS = length of hospital stay; VAS = visual analog scale; BMI = body mass index.

**Table 2:** Studies assessing the use of bowel preparation in vaginal surgery.

Study (reference)	Regimen of BP compared (group size/no. of patients)		Outcomes of interest and results
Ballard et al. [14]	MBP-saline enema (75)	No MBP (75)	No difference in surgeons' assessment of surgical field No difference in blood loss Higher rates of hunger pains, abdominal cramps, abdominal fullness and bloating, and decreased patients' satisfaction in MBP group

Adelowo et al. [13]^a^	MBP-oral magnesium citrate + NaP enema (71)	MBP-NaP enema (77)	Greater patients' overall discomfort and negative preoperative side effects, such as abdominal cramping or pain, bloating or swelling, embarrassment, weakness, dizziness, and fecal incontinence in oral and rectal MBP group Better overall evaluation of the surgical field at initial port placement in combined MBP group. No difference at the conclusion of the surgery Better visualization of the uterus in the combined MBP group No significant difference in visualization of adnexal structures between groups No difference in first bowel movement or initial passage of flatus

Deng et al. [15]	MBP-NR (60)	No MBP (60)	Higher rates of fecal contamination of surgical field in MBP group Higher rates of nausea, vomiting, abdominal distension, fatigue, and palpitation in MBP group

Tayyab et al. [16]	MBP-2 saline enemas (30)	No MBP (30)	No difference in postoperative nausea, vomiting, and anal irritation

MBP = mechanical bowel preparation; NaP = sodium phosphate. ^a^Laparoscopic or robotic surgical correction of apical prolapse.
